# Impact of electronic portal image quality on Elekta AQUA^®^ collimator isocenter

**DOI:** 10.1002/acm2.13934

**Published:** 2023-03-01

**Authors:** Craig A. J. Norvill

**Affiliations:** ^1^ Genesis Care Perth Australia

**Keywords:** AQUA, EPID, Isocenter

## Abstract

**Purpose:**

The collimator radiation isocenter position determined in AQUA^®^ v3.0 [Elekta AB, Stockholm, Sweden] software test “MLC Leaf and Jaw Position” was independently validated using an in‐house MATLAB [Natick, MA: The MathWorks Inc.] script.

**Methods:**

The AQUA test determines radiation isocenter using the mean field center of nine 4 cm × 4 cm electronic portal imager (EPID) exposures at equidistant collimator angles. Impact of EPID image quality on AQUA reported isocenter for thirteen Elekta linear accelerators with Agility MLC heads were evaluated.

**Results:**

Of the thirteen, three had visually and quantitatively identifiable artifacts. For the ten good EPID's there was a systematic 0.25 mm offset of the MATLAB calculated mean field center relative to AQUA in the X‐axis and Y‐axis. This corresponds to one image pixel and was found to be due to differences in software co‐ordinate convention. After subtracting this offset there was no significant difference in AQUA and MATLAB calculated isocenter.

**Conclusions:**

For the three machines with poor image quality there was a demonstrated variation in AQUA calculated field center and therefore radiation isocenter relative to MATLAB. Restricting the region of interest (ROI) in AQUA software to only the irradiated section of the EPID brought AQUA and MATLAB result for these three machines into agreement.

## 1 INTRODUCTION

Accurate multi‐leaf collimator (MLC) positioning is a key component of any radiation oncology QA program and multiple publications reference recommendations on testing methods, frequency and tolerances.^1^, ^2^, ^3^ AQUA^®^ v3.0 [Elekta AB, Stockholm, Sweden] software is a program for comprehensive quality assurance (QA) testing, scheduling and database recording in radiation oncology departments. The AQUA test “MLC Leaf and Jaw Position LinacConnect” (hereafter referred to as “MLC QA”) uses an electronic portal imaging device (EPID) to determine the absolute position of linear accelerator (linac) MLC leaves relative to the collimator radiation isocenter.^4^ EPID's have been demonstrated to allow rapid image acquisition, processing and evaluation of all MLC leaves over multiple off axis positions in both static and dynamic modes.^5^, ^6^, ^7^, ^8^ Recommendations for management of an EPID image quality program are discussed in several publications.^9^, ^10^

The test method applied in AQUA is described in the publication by Le Tourneau et al.^11^ for an Elekta linear accelerator and iViewGT^TM^ [Elekta AB, Stockholm, Sweden] EPID.^12^, ^13^ The MLC QA test requires the EPID panel center to be offset 13 cm toward the gantry relative to the machine isocenter. This is necessary such that all MLC leaves can be captured within one static EPID and two collimator (0°, 180°) positions. The megavoltage (MV) radiation isocenter of the collimator is first determined by finding the radiation center of nine 4 cm × 4 cm images, acquired at equidistant collimator rotations (40‐degree increments). The radiation center of each field are then calculated using an iterative thresholding algorithm,^14^ and an AQUA reported radiation isocenter determined by fitting a circular function to the nine field centers.

The radiation isocenter co‐ordinate is then subsequently used as the absolute reference point for calculating MLC leaf positions, with a further correction factor based on the mean positional difference (collimator 0° to 180°) of leaf pairs 17–24.^11^ Error in calculation of the radiation isocenter will translate into a systematic error in determination of all MLC leaf positions.

The AQUA test guide^4^ states that the region of interest (ROI) applied when analyzing the collimator rotation images may be restricted, which can be useful for excluding some image artifacts. EPID image quality issues may be due to factors such as background (offset) correction, gain calibration, background ghosting (watermark), pulse synchronization, bad pixels and sub‐panel contrast.^12^

The purpose of this study was to independently validate the calculated radiation isocenter across multiple Elekta linear accelerators using the same fields as captured for AQUA MLC QA, and evaluate the impact of EPID image quality on the test result. Furthermore, it aimed to assess the impact on calculated radiation isocenter and reported MLC position in AQUA by applying a restricted ROI.

## 2 METHODS

### 2.1 Qualification of EPID panels

**FIGURE 1:**
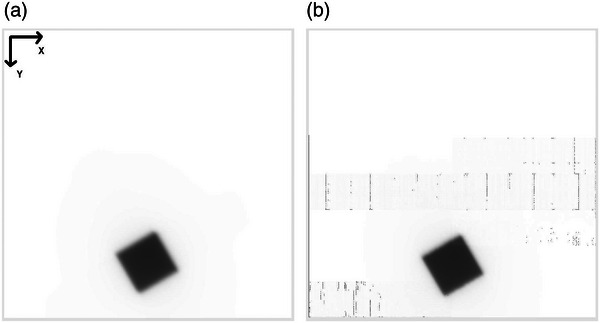
Example of a visually evaluated (a) good image (machine D) and (b) poor image (machine G).

An example MLC QA test image is given in Figure 1. DICOM images exported from the iViewGT^TM^ system are a 1024 × 1024 matrix of nominally 0.25 mm pixel spacing at the machine isocenter plane. X and Y axes increase from the top‐left corner of the image. Images are a default 16‐bit unsigned integer grayscale format, with zero representing black (maximum signal) pixels and 65 535 representing white (zero signal) pixels.

Of the thirteen linear accelerators in this study, all are calibrated during routine annual EPID preventative maintenance, and may be calibrated more frequently based on user feedback or routine testing. The iView panels in this study were not used for clinical patient treatment setup, and were only used as a tool for machine QA or research. Qualitative assessment was performed by visual assessment of the collimator isocenter images without any contrast enhancement or other processing applied. Machines that contained images with identifiable subpanels or bad pixels (random or lines) were classified as “poor” while machines where artifact was not visually identifiable were defined as “good”. The nine radiation isocenter images for each machine were imported to MATLAB [*R2017a*. Natick, MA: The MathWorks Inc.]. A 1‐pixel border was trimmed from the image to account for edge artifacts. Images were quantitatively assessed by calculating signal to noise ratio (SNR) of the unexposed region of the panel. The unexposed region was defined as the area outside an 8.5 cm × 8.5 cm mask, applied over the ROI containing the irradiated 4 cm × 4 cm field. The mean (*μ_x_*) and standard deviation (*σ_x_*) of included pixel intensities were calculated for each image, and SNR calculated as described in Equation (1). Given the signal was sampled in the unexposed region of the panel, the metric was not strictly a measure of SNR, however was still considered a useful metric for image quality comparison across machines.

(1) $$\mathrm{SNR}\;=\frac{\mu_{x}}{\sigma_{x}}\;$$

### 2.2 MATLAB field center calculation

A script was written in MATLAB to independently calculate the radiation isocenter. Details are provided in the Appendix. Each of the nine DICOM images were imported into MATLAB and converted from 16‐bit greyscale to a binary format using the “imbinarize” command. The command uses Otzu's method to auto‐threshold black and white pixels based on minimizing intraclass variance.^15^ The same mask described in section 2.1 was applied to the DICOM images. Regions within the mask ROI were assigned a value of one, and all other pixels outside the ROI assigned a value of zero. The mask was matrix multiplied with the inverted binary image, such that all pixels outside the mask ROI were set to zero. The MATLAB “regionprops” command was used to return the X‐axis and Y‐axis centroid co‐ordinates for each field. In a beams‐eye view, the X‐axis represents left to right when viewing the gantry from foot of the treatment couch, while the Y‐axis represents electron gun to target direction. Pixel co‐ordinates for each machine were converted to millimeters using the machine specific pixel scale factor.^13^

**FIGURE 2:**
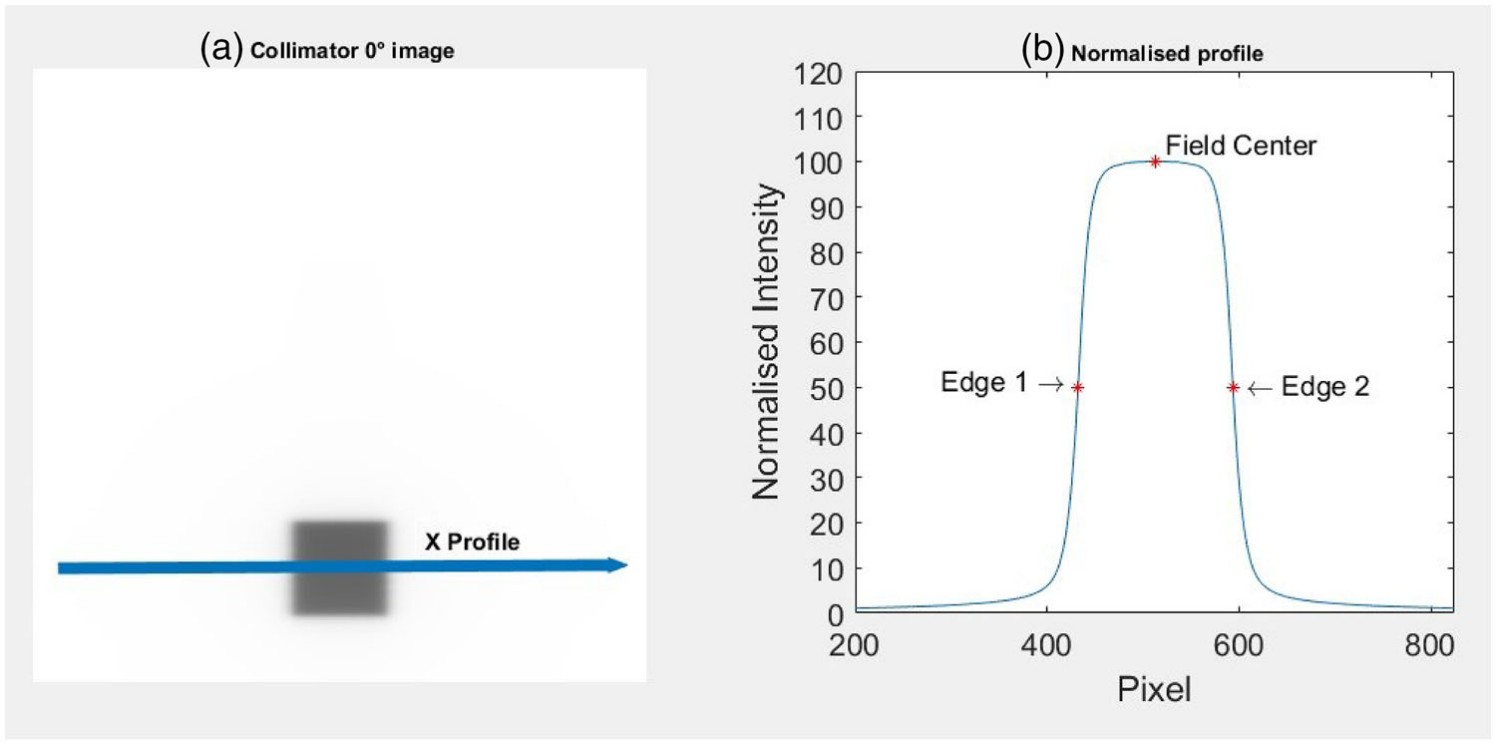
Independent method to calculate field center by (a) extraction of profile across collimator 0° image and (b) extraction of normalized 50% penumbra field edges.

To validate the binary method, field center was also calculated without binarizing images. This was done only for the collimator zero‐degree images for each machine, so that the irradiated field edges were parallel to the image row/columns. The image pixel intensities were inverted and twenty profiles acquired in the X‐axis and Y‐axis across the center 5 mm of the field. The profiles were averaged and then normalized to the field center. Field edges were defined as the position corresponding to 50% of the field center pixel intensity, using a linear interpolation of points within a 60%‐40% region of the penumbra (Figure 2). The field center for each profile was calculated as the mean of the first and second field edge. A paired two tailed t‐test was applied to evaluate whether any statistical difference in mean field center existed for the MATLAB binary and field edge methods.

### 2.3 Comparison of MATLAB and AQUA isocenter

For each machine, the AQUA reported field center co‐ordinates (X‐axis, Y‐axis) for the nine collimator angle projection images were averaged to give a mean field center co‐ordinate. The AQUA calculated mean field center is here referred to as “AQUA Mean”. In addition to the X‐axis and Y‐axis field center's, AQUA reports the co‐ordinate that is derived from the circular fitting function previously mentioned. This co‐ordinate is used by AQUA as the radiation isocenter and is hereafter referred to as “AQUA ISO”. AQUA co‐ordinates for each machine were converted to millimeters using the same machine specific pixel scale factors used in the MATLAB calculation.

The same AQUA test images for each machine were processed with the MATLAB binary script. Correspondence with the vendor confirmed that AQUA references the top‐left pixel of the image as co‐ordinate [0,0], while MATLAB defines this as [1,1]. Therefore, 1‐pixel was subtracted from the MATLAB calculated [X, Y] results. MATLAB calculated mean field center co‐ordinate for each machine were compared to the AQUA (ISO and Mean) values.

### 2.4 Restricted AQUA region of interest

The impact of restricting the ROI in AQUA was assessed by applying the same ROI mask in MATLAB and AQUA to each image. The test configuration of each machine in AQUA was modified with restricted ROI co‐ordinates of [340,680,680,1020]. These correspond to the row and column position of the top‐left and bottom‐left corner of the ROI. Each set of images was then re‐run in AQUA, and the radiation isocenter results compared to those using the full unrestricted image. The impact of image artifact on reported MLC leaf positions was further assessed by comparing AQUA reported MLC positions for restricted and un‐restricted ROI test runs. A total of 800 data points (2 leaf‐banks, 5 off‐axis positions, and 80 leaves per bank) were recorded per machine.

## 3 RESULTS

### 3.1 Qualification of EPID panels

**FIGURE 3:**
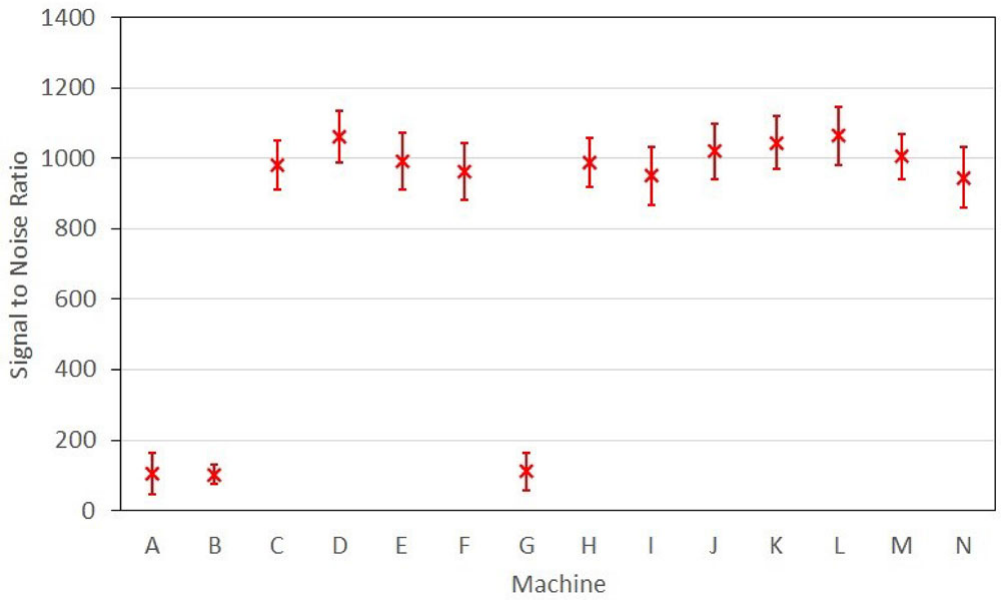
Signal to noise ratio of non‐exposed pixels averaged over the nine collimator rotation images. Error bars indicate 1 standard deviation.

Calculated SNR for each of the machines is shown in Figure 3. Error bars show standard deviation of calculated machine SNR over the nine exposures. SNR for machines A, B and G ranged between 103 to 111 and for all other machines ranged from 946 to 1064. Machines A, B and G showed clear image artifact and/or visible subpanels and were classified as poor images, while all other machines displayed no obvious qualitative issues. Figure 1 shows examples of images acquired on machine C (a good image) and machine G (a poor image).

### 3.2 MATLAB field center calculation

**FIGURE 4:**
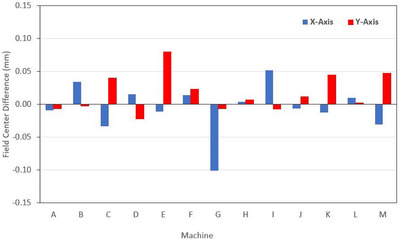
Difference in MATLAB calculated field center (Collimator 0° image) for binarize method relative to field edge penumbra method.

Figure 4 shows the difference in MATLAB calculated field center for the collimator zero images, calculated using the binary method relative to the field edge method.

For the thirteen machines, mean difference in field center co‐ordinate for binary and field edge methods on the X‐axis was −0.01 mm [0.04 mm], which was not significant at the 0.05 level, *t*(13) = 0.56, *p* = 0.58. Similarly for the Y‐axis, mean difference of 0.02 mm [0.03 mm] was not significant, *t*(13) = −2.00, *p* = 0.07. Maximum difference in field center co‐ordinates was 0.1 mm, however this was for machine G which was shown to have poor image quality.

### 3.3 Comparison of MATLAB and AQUA isocenter

For the good panels (excepting machines A, B, and G), the mean difference in isocenter co‐ordinate between AQUA ISO and AQUA Mean methods was 0.02 mm [0.03 mm] for the X‐axis and −0.02 mm [0.03 mm] for the Y‐axis. Maximum difference was −0.07 mm (machines C and F). Of the machines with image artifact, machine G had the largest difference between AQUA ISO and AQUA Mean results, being 0.58 mm in the X‐axis and 0.21 mm in the Y‐axis.

**FIGURE 5:**
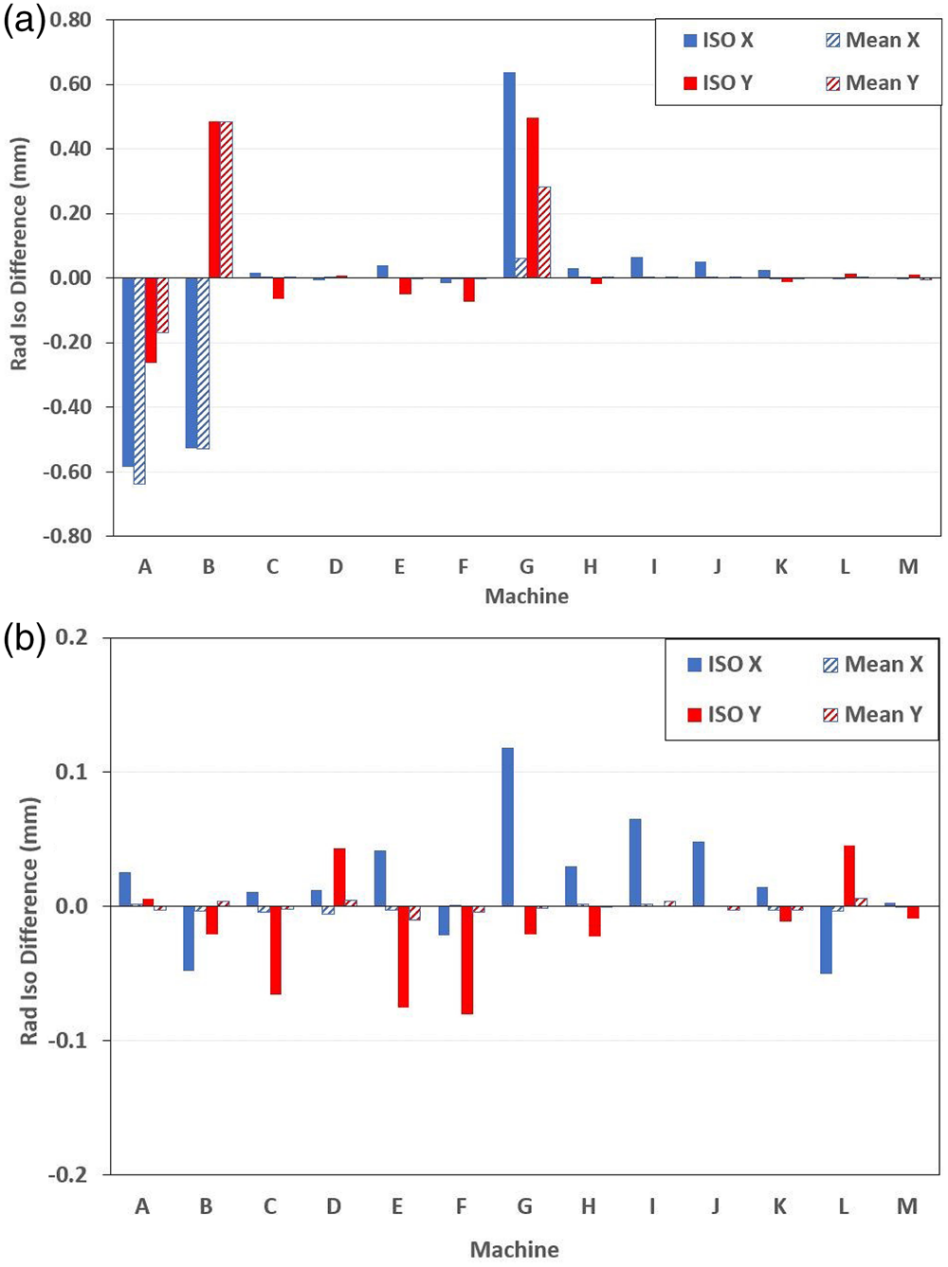
MATLAB calculated radiation isocenter relative to AQUA ISO and AQUA Mean for (a) default AQUA test image ROI and (b) restricted AQUA image ROI (difference axis rescaled for visualization). ROI, region of interest.

The difference in MATLAB calculated isocenter co‐ordinate (mean of nine image field center's) relative to the AQUA ISO and AQUA Mean result is given in Figure 5a. For good panels, mean difference in calculated MATLAB isocenter relative to AQUA Mean was 0.00 mm [SD < 0.00 mm] in both X‐axis and Y‐axis. And for good panels, MATLAB relative to AQUA ISO result was 0.02 mm [0.03 mm] and −0.02 mm [0.03 mm] for X‐axis and Y‐axis respectively. For the machines A, B and G with visible image artifact, the difference between MATLAB and AQUA isocenter varied between −0.58 and +0.64 mm.

**FIGURE 6:**
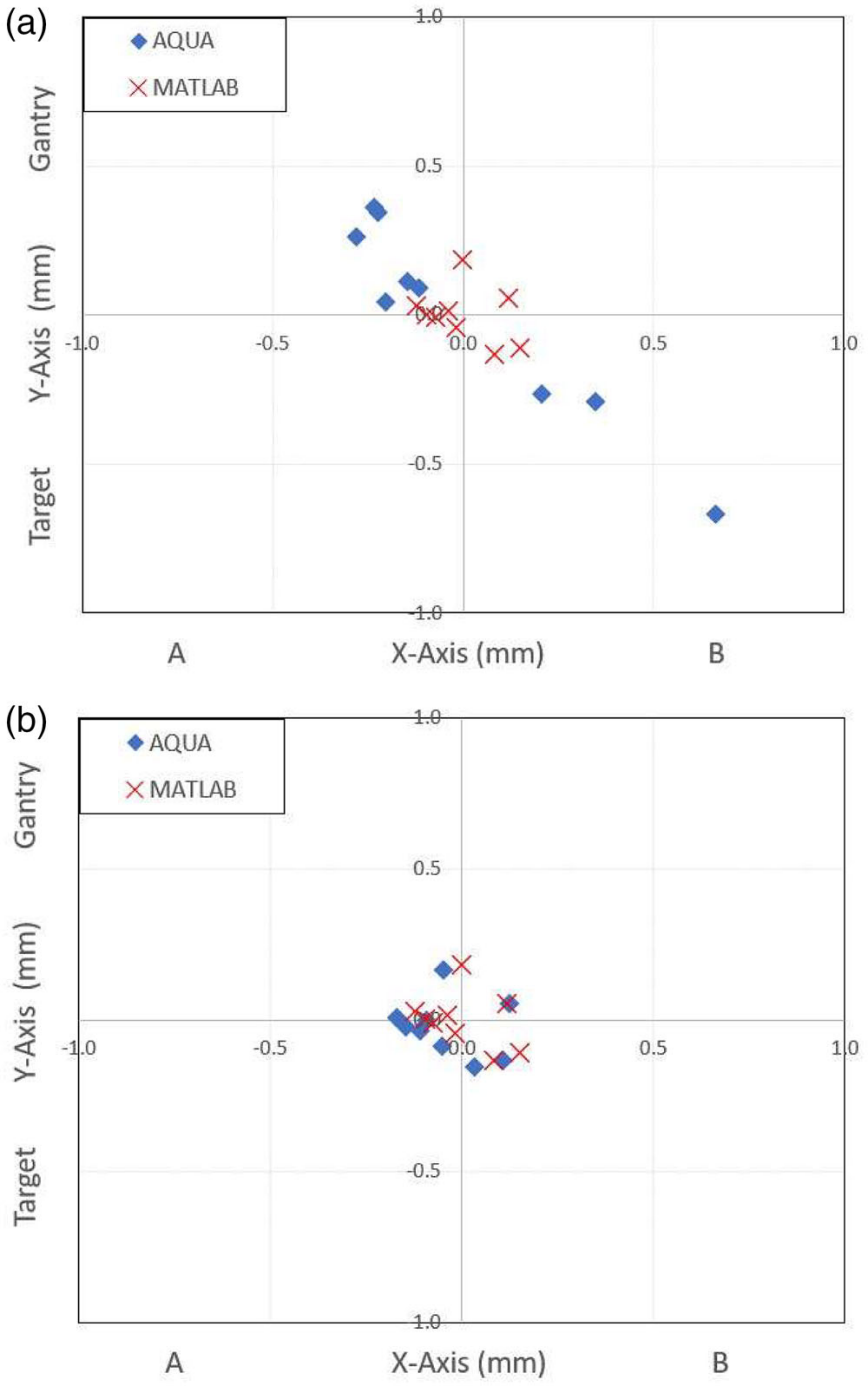
AQUA and MATLAB calculated field center relative to mean of nine image field centers. AQUA data calculated using (a) default AQUA ROI settings and (b) using restricted ROI settings. ROI, region of interest.

Taking Machine B as an example, Figure 6a shows the AQUA and MATLAB field center co‐ordinates relative to the respective calculated isocenter. The impact of image artifact on the calculation of the AQUA field centers is evident, with the distribution not radially arranged around the mean value.

### 3.4 Restricted AQUA region of interest

For good image panels, difference in AQUA ISO for the unrestricted and restricted ROI was a maximum of 0.05 mm (machine L), with a mean difference of 0.01 mm [0.02 mm] and 0.00 mm [0.02 mm] for X and Y axes.

The difference between MATLAB and AQUA (restricted ROI) calculated radiation isocenter are given in Figure 5b (Y‐axis rescaled so as to visualise data columns). Restricting the AQUA ROI improved MATLAB to AQUA agreement for all the machines (A, B, and G) with image artifact. For all machines including poor image panels, averaged MATLAB calculated isocenter relative to the AQUA Mean was 0.00 mm [SD < 0.00 mm] for both axes, and MATLAB relative to AQUA ISO was 0.02 mm [0.05 mm] and −0.02 mm [0.04 mm] for the X and Y axes. In a paired t‐test, MATLAB isocenter relative to AQUA ISO was not significant for the X‐axis, *t*(13) = 1.54, *p* = 0.15 or Y‐axis, *t*(13) = 1.44, *p* = 0.17.

Figure 6b shows the resultant change in AQUA field center relative to the AQUA ISO co‐ordinate for Machine B. Maximum root mean square (RMS) field offset relative to the isocenter for this machine decreased from 0.94 to 0.17 mm, with the mean field RMS offset decreasing from 0.38 mm [0.25 mm] to 0.14 mm [0.03 mm].

**FIGURE 7:**
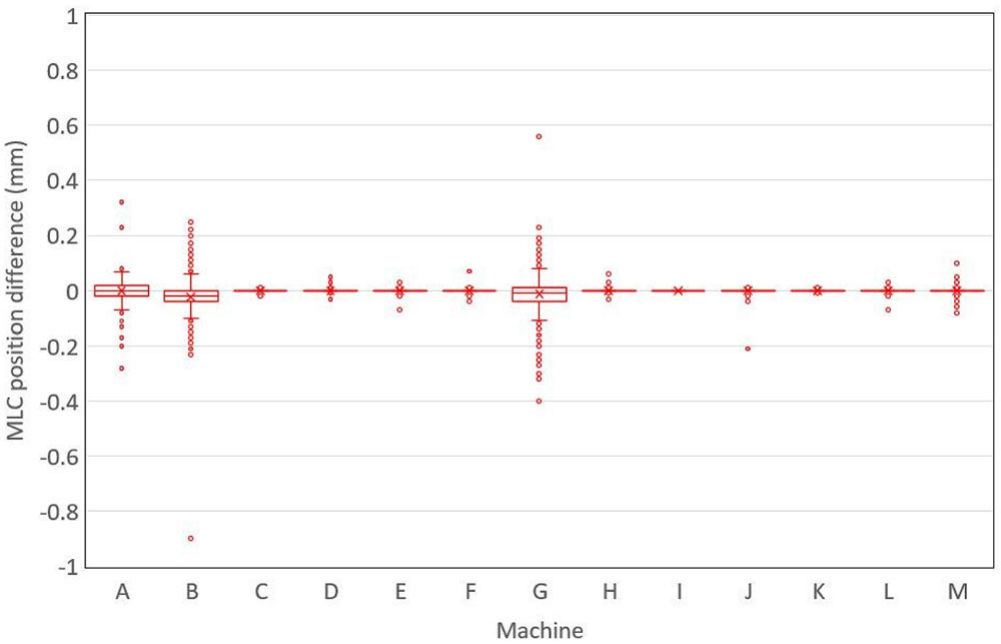
Box‐plot of AQUA reported MLC leaf position difference (restricted – unrestricted ROI) for all leaf positions (*n* = 800 leaf positions per machine). MLC, multi‐leaf collimator; ROI, region of interest.

Figure 7 shows a box plot of the difference in AQUA reported MLC leaf position for restricted relative to unrestricted ROI test runs. The “difference” axis is scaled to ±1 mm for visualization, however there were three outliers exceeding this range (−0.9 mm and −1.1 mm for machine B, and +3.0 mm for machine G). For the poor panels, median differences were 0.00, −0.02 and −0.01 mm for machines A, B, and G respectively, with the minimum and maximum box‐plot whiskers falling between ±0.1 mm. The proportion of data points (*n* = 800) exceeding ±0.1 mm difference (max range of whiskers for poor panels) between restricted and unrestricted ROI test runs was 1.4%, 7.6%, and 10.4%. For all good panels, median difference was 0.00 mm with first and third quartiles being equivalent to the median value of 0.00 mm, and a maximum outlier difference for a single leaf position of −0.21 mm (machine J).

## 4 DISCUSSION

Of the thirteen machines sampled, three had visually evident image artifacts which was shown to be consistent with the calculated SNR. The physical location of the noisy pixels was different in each of these cases, and was largely due to the location of sub‐optimal sub‐panels. Of the three EPIDs with image artifacts, all had previous calibrations performed without significant improvement to image quality. Panels weren't calibrated immediately prior to data collection as this was considered not to be indicative of normal operating performance.

For the machines without image artifact, the MATLAB binary method of calculating radiation isocenter co‐ordinates gave an identical result (to two significant figures) relative to AQUA Mean. Both MATLAB and AQUA Mean radiation isocenter were calculated using the same method (mean of all field center's), therefore indicate consistency in two separate software programs for extraction of image field center co‐ordinate. The further processing applied in AQUA ISO only made small (max 0.07 mm) differences when compared to the AQUA Mean result. The AQUA ISO result however can be significantly impacted by image artifact, with differences of up to 0.6 mm on the poor images. As shown in Figure 6a. the non‐radial distribution of the field center's on the poor images would be a likely explanation as to why AQUA has difficultly in applying a meaningful circular fitting function in these cases.

Masking image pixels outside the ROI in AQUA had negligible effect on good images, but improved the MATLAB to AQUA agreement significantly for the poor images. After application of the restricted ROI in AQUA software there was no significant difference between MATLAB and AQUA ISO result irrespective of image artifact.

For the good image panels, there was no significant difference in reported MLC leaf position for the AQUA restricted and AQUA unrestricted ROI test run of the same images, which is reasonable given the negligible change in AQUA reported collimator isocenter. For the poor image panels a substantial percentage of reported leaf positions were greater than 0.1 mm different, with two being over 1 mm. One millimeter is a commonly recommended tolerance for MLC leaf position accuracy,^1^ and the AQUA test will fail if a single leaf position error exceeds the set tolerance. Therefore, the variation in reported MLC position for the poor panels could be sufficient to change the test result, particularly for a leaf position error that was already close to tolerance.

This study showed that to achieve an accurate AQUA test result for radiation isocenter it is important to use an EPID without significant image artifacts. This should be implemented via a regular qualitative and/or quantitative review of panel performance. However, for older or damaged panels there are limits to which the image may be corrected via re‐calibration, and replacement of the EPID can be an expensive operational cost. While non‐optimal panels may still be suitable for machine performance monitoring, applying a ROI in AQUA software for the MLC Leaf and Jaw Position LinacConnect test is recommended to reduce the impact of image artifacts.

## 5 CONCLUSION

EPID image artifact was shown to impact the result of radiation isocenter calculation in AQUA software. Applying a ROI in AQUA software can minimize the impact of poor image quality. Regular visual and/or quantitative evaluation of MV portal images is also recommended such that an accurate definition of radiation isocenter may be determined.

## AUTHOR CONTRIBUTIONS

This work (measurement, analysis and write up) was performed by the author listed.

## CONFLICT OF INTEREST STATEMENT

The author declares no conflicts of interest.

## Data Availability

The data that support the findings of this study are available from the corresponding author upon reasonable request.

## 1

%For each image (1‐9), convert to binary and invert, then apply mask of zeros for area outside ROI. Return the image centroid pixel X, Y co‐ordinate to variable RadISO. Repeat for all images.

ImageDir = uigetdir();

ImageFiles = dir (‘*.DCM’);

RadCenter = zeros(9:2);

for img_num = 1:9

Filename = ImageFiles(img_num).name;

AQUAImage = dicomread(Filename);

mask = zeros(size(AQUAImage));

mask(680:1020,340:680) = 1;

AQUAImage_binary = imbinarize(AQUAImage);

AQUAImage_binary = imcomplement(AQUAImage_binary);

AQUAImage_binary = AQUAImage_binary .* mask;

stats(img_num) = regionprops(AQUAImage_binary,‘Centroid’);

RadISO(img_num,:) = stats(img_num).Centroid;

end

csvwrite(‘RadISO_Result.csv’,RadISO);
